# RGB-FIR Multimodal Pedestrian Detection with Cross-Modality Context Attentional Model

**DOI:** 10.3390/s25133854

**Published:** 2025-06-20

**Authors:** Han Wang, Lei Jin, Guangcheng Wang, Wenjie Liu, Quan Shi, Yingyan Hou, Jiali Liu

**Affiliations:** 1 School of Transportation and Civil Engineering, Nantong University, Nantong 226019, China; hanwang@ntu.edu.cn (H.W.); 2433320008@stmail.ntu.edu.cn (L.J.); wanggc@ntu.edu.cn (G.W.); lwj2014@ntu.edu.cn (W.L.); sq@ntu.edu.cn (Q.S.); 2 Target Key Laboratory of Cognition and Application Technology, Aerospace Information Research Institute, Chinese Academy of Sciences, Beijing 100094, China; houyy@aircas.ac.cn; 3 Key Laboratory of Network Information System Technology, Aerospace Information Research Institute, Chinese Academy of Sciences, Beijing 100094, China; 4 School of Intelligent Manufacturing and Information, Jiangsu Shipping College, Nantong 226010, China

**Keywords:** cross-modality context attentional model, RGB-FIR multimodal YOLO, pedestrian detection, multi-level fusion strategy

## Abstract

Pedestrian detection is an important research topic in the field of visual cognition and autonomous driving systems. The proposal of the YOLO model has significantly improved the speed and accuracy of detection. To achieve full day detection performance, multimodal YOLO models based on RGB-FIR image pairs have become a research hotspot. Existing work has focused on the design of fusion modules after feature extraction of RGB and FIR branch backbone networks, achieving a multimodal backbone network framework based on back-end fusion. However, these methods overlook the complementarity and prior knowledge between modalities and scales in the front-end raw feature extraction of RGB and FIR branch backbone networks. As a result, the performance of the backend fusion framework largely depends on the representation ability of the raw features of each modality in the front-end. This paper proposes a novel RGB-FIR multimodal backbone network framework based on a cross-modality context attentional model (CCAM). Different from the existing works, a multi-level fusion framework is designed. At the front-end of the RGB-FIR parallel backbone network, the CCAM model is constructed for the raw features of each scale. The RGB-FIR feature fusion results of the lower-level features of the RGB and FIR branch backbone networks are fully utilized to optimize the spatial weight of the upper level RGB and FIR features, to achieve cross-modality and cross-scale complementarity between adjacent scale feature extraction modules. At the back-end of the RGB-FIR parallel network, a channel-space joint attention model (CBAM) and self-attention models are combined to obtain the final RGB-FIR fusion features at each scale for those RGB and FIR features optimized by CCAM. Compared with the current RGB-FIR multimodal YOLO model, comparative experiments on different performance evaluation indicators on multiple RGB-FIR public datasets indicate that this method can significantly enhance the accuracy and robustness of pedestrian detection.

## 1. Introduction

The unmanned vehicles (UVs) based on multi-sensor fusion have begun to be applied in fixed route areas such as industrial parks, school campuses, and scenic areas [1,2]. Pedestrian detection based on visual cognition is an important research problem faced by autonomous vehicles in achieving automatic obstacle avoidance function. Its main task is to identify pedestrian targets in vehicle-mounted camera images and regress their confidence, position, and scale information [3,4]. However, due to the influence of shooting distance, angle, and especially lighting conditions [5,6,7], building a pedestrian detection model with high accuracy and robustness has become a challenging task.

**Figure 1:**
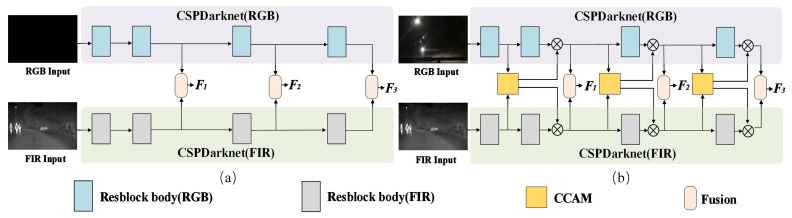
Frameworks of RGB-FIR multimodal YOLO backbone network for autonomous driving systems. (**a**) Current network with back-end fusion strategy; (**b**) Proposed network with multi-level fusion strategy.

Under good lighting conditions, RGB images can provide color and texture information. Advanced models such as Faster R-CNN [8], SSD [9], and YOLO [10] utilize this information to generate appearance description features of pedestrian targets, accurately identifying them. However, the sharp decrease in contrast of RGB images in low light environments will significantly degrade the performance of the YOLO pedestrian detection model [11,12]. Far-infrared (FIR) images can describe the heat distribution of a scene, and pedestrian targets typically exhibit high brightness values in FIR images and form a sharp contrast with the surrounding environment. It is worth noting that, in this paper, FIR refers to far-infrared thermal imaging, which is distinct from the commonly used “FLIR” (Forward-Looking Infrared). FIR here represents the broader spectral band of far-infrared sensing used in multimodal vision systems. In addition, FIR images are not affected by glare, and the shape and contour information of the human body can effectively represent pedestrian targets [13,14]. Therefore, FIR cameras are widely used in nighttime pedestrian target detection tasks, as shown in Figure 1a.

In order to reduce the impact of lighting conditions and obtain robust pedestrian detection results, researchers have made some attempts to enhance pedestrian detection by fusing RGB and FIR image features [15,16,17]. The simple structure, high accuracy, and good real-time performance of the YOLOv5 model [18] has spurred research of the RGB-FIR multimodal YOLO pedestrian detection network model; Models such as YOLO-CMAFF [19], Dual-YOLO [20], and MAF-YOLO [21] have validated the effectiveness of RGB-FIR modality complementary in pedestrian detection. Current RGB-FIR multimodal YOLO pedestrian detection models all adopt the RGB-FIR parallel backbone network structure, which uses an effective multimodal feature fusion module for single-modality RGB and FIR output features at the bottom, middle, and upper scales of the parallel backbone network. By implementing the back-end feature fusion framework of the RGB-FIR parallel backbone network, different scales of RGB-FIR multimodal fusion features are obtained [22], to enhance the representation ability of pedestrian targets.

Current RGB-FIR parallel backbone networks [19,20,21] are based on a back-end fusion strategy, as shown in Figure 1b. Each branch adopts the same CSPDarknet structure as the YOLOv5 backbone network, which consists of four Resblbockbody connected in series. After extracting RGB and FIR features, RGB-FIR multimodal feature fusion is performed, whose effectiveness for pedestrian target representation depends on the design scheme of the feature fusion module and the representation ability of RGB and FIR single-modality original features. When RGB and FIR features cannot effectively represent pedestrian targets, RGB-FIR fused features have difficulty enhancing their representation. The backbone network of the YOLO model series [18,23,24,25] is composed of low-level, intermediate, and high-level multiscale feature extraction modules. With the transition of features from bottom to top and local to global, the spatial resolution size of the output feature map gradually decreases, and the receptive field gradually expands. During this process, the spatial importance information of some low-level local effective features is gradually diluted until they disappear during iteration. This has led to pedestrian target feature drift and even loss in the YOLO model during the feature extraction process from bottom to top for RGB images in low light environments.

**Figure 2:**
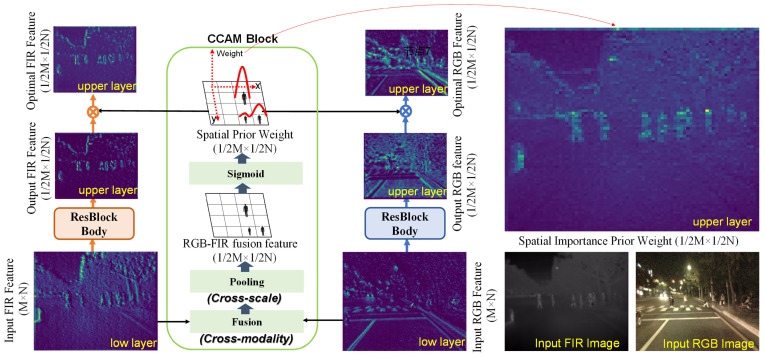
Explanation of the visualization motivation of CCAM.

If we utilize the effective human body description features at the bottom layer to generate prior weights for the spatial position of upper layer pedestrian features during the iterative process of features from the bottom to the top, and use this weight value to adjust and correct the spatial position importance of adjacent upper layer RGB and FIR iterative features, it can effectively prevent the possibility of pedestrian feature spatial position drift or even loss during the feature extraction process of YOLO model from bottom to top. Inspired by the above ideas, this paper proposes a novel cross-modality context attentional model (CCAM) in the front-end of RGB-FIR multimodal parallel backbone network. Using the low layer fusion features to generate the spatial importance prior weights for upper layer features, and using this prior weight value to adjust and correct the spatial importance of the upper layer RGB and FIR iterative features, cross-modality and cross-scale feature complementarity and fusion are achieved. The explanation of its visual design motivation is shown in Figure 2. The main contributions of this paper are as follows:

(1) We propose an RGB-FIR multimodal YOLO backbone network framework based on a multi-level fusion strategy, as shown in Figure 1d, consisting of an RGB-FIR parallel backbone network, a cross-modality contextual attention model, and a multimodal feature fusion module.
(2) A CCAM model is designed for the front end of the RGB-FIR parallel backbone network to achieve cross-modality and cross-scale complementarity between adjacent scale features.
(3) With comparative experimental results, we analyze the performance of feature fusion strategies at different positions of the RGB-FIR parallel backbone network for the multimodal pedestrian detection models.

## 2. Related Work

### 2.1. Pedestrian Detection Model Based on RGB Images

The statistical model characterizes targets through manually designed feature operators. Among the commonly used feature operators, Haar [26], HOG [27], CO-HOG [28], LBP [29], and SIFT [30] characterize the local contrast change law of the image, changes in local contours of the human body, combined distribution law of multiple local contour changes in the human body, change law of local texture information, and features that represent local scale invariance, respectively. The above operators manually extract features based on the pedestrian features through different design data structures. The statistical pedestrian detection model has low complexity and is easy to implement [31,32]. Unlike statistical pedestrian detection models, deep learning methods based on CNNs [33,34] learn and obtain features with stronger representation and generalization capabilities hidden within the data. Ross et al. [35] created R-CNN by combining region-based suggestion technology with a CNN. Furthermore, Ren et al. [36] added an RoI pooling layer in the Fast R-CNN model, which is faster and has better detection performance.

Joseph et al. [37] proposed a YOLO pedestrian detection model achieving higher accuracy by dividing the image into grids and predicting whether each grid contains targets, along with their positions. The continuous upgrading of the YOLO model structure results in increased performance of pedestrian detection [38]. For small target detection tasks in complex scenes, Liu et al. [39] proposed single-shot multi-box detector (SSD), which applies convolutional filters on each feature layer to simultaneously predict the target’s category and bounding box, seeking a balance between speed and accuracy. This was followed by a series of optimized and upgraded models such as DSSD [40], FSSD [41], and DSOD [42].

In recent years, transformer-based architectures have also demonstrated impressive performance in pedestrian detection. Yuan et al. [43] investigated the effectiveness of deformable vision transformers for single-stage pedestrian detection and proposed a spatial and multi-scale feature enhancement module to strike a balance between speed and accuracy. Their method outperformed both single-stage and two-stage detectors on the Caltech and CityPersons datasets, particularly excelling in heavy occlusion scenarios while using fewer parameters. Similarly, Wu et al. [44] proposed the RT-DETR-improved model, which integrates HiLo attention and a nonlinear feature fusion module to enhance performance across complex urban scenes. Their model also introduced a novel loss function, InnerMPDIoU, improving detection accuracy and robustness, especially in crowded environments, with notable gains in mAP, accuracy, and recall on the CityPersons dataset. Although these transformer-based approaches have achieved remarkable results, they are predominantly designed for single-modality inputs such as RGB images. In low-light or adverse weather conditions, single-modality detectors may suffer from limited feature representation and degraded detection performance. This motivates the need for multimodal pedestrian detection frameworks, which integrate complementary information from both RGB and FIR modalities to enhance detection robustness and accuracy under challenging environments.

### 2.2. RGB-FIR Multimodal Pedestrian Detection Model

Hwang et al. [45] proposed the ACF pedestrian detector for FIR images, using HOG features to expand the aggregation channel features, marking the beginning of multimodal pedestrian detection technology. Subsequently, Xu et al. [46] proposed a two-stage cross-modal learning framework that models the pixel value mapping relationship between RGB and FIR images and transfers the learned features to another deep learning network, effectively improving detection accuracy. Zhang et al. [47] utilized the interactive nature of multispectral input sources to solve the problem of contradictory appearance in multimodal data and improved the accuracy and robustness of pedestrian detection by encoding inter-modal correlations through feature hierarchy and attention modules. Zhou et al. [48] proposed MB-Net to address the problem of modal imbalance in multimodal pedestrian detection. The image fusion network STDFusionNet [49] can effectively fuse the features of two modalities and reconstruct the desired results.

Due to the simple structure, high accuracy, and good real-time performance of YOLOv5, there has been much optimization research on the RGB-FIR multimodal YOLO pedestrian detection network model since 2021. Xue et al. [21] used CSPdarknet to construct an RGB-FIR parallel backbone network and designed MAF, a channel spatial attention fusion model for multimodal feature fusion based on the original output features of the RGB and FIR scales. Similarly, Fang et al. [19] designed a cross-modality attention fusion model, CMAFF, for RGB and FIR raw output features of the same scale in the RGB-FIR parallel backbone network, and obtained RGB-FIR fusion features at the bottom, middle, and high scales. In the RGB-FIR parallel YOLOv7 backbone network, Bao et al. [20] used the inception module containing multiple receptive fields to conduct multiscale fusion and shuffle for RGB and FIR features.

## 3. Proposed Method

### 3.1. Cross-Modality Context Attentional Model (CCAM)

**Figure 3:**
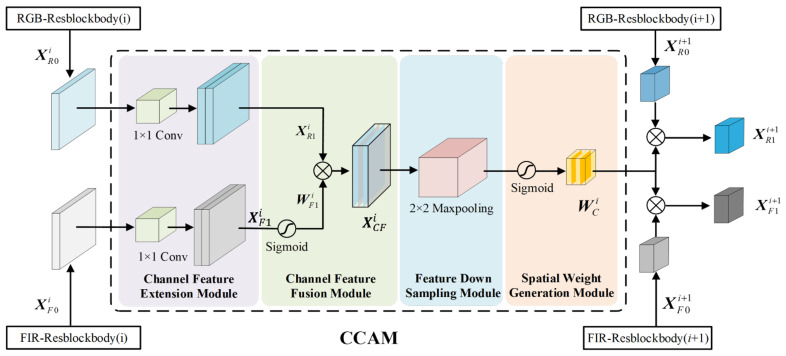
Framework of CCAM.

The proposed CCAM uses the features of the *i*-th Resblockbody to generate spatial weights for the output features of the i + 1th Resblockbody. As shown in Figure 3, the proposed CCAM model consists of four modules in series: Channel Feature Extension (CFE), Channel Feature Fusion (CFF), Feature Down Sampling (FDS), and Spatial Weight Generation (SWG).

(1) Channel feature extension module: The CFE module is shown in the purple section of Figure 3. The CFE module is used to expand the number of feature channels of the *i*-th Resblockbody to be the same as the number of feature channels of the *i* + 1th Resblockbody. Let the output features of the *i*-th Resblockbody in the RGB and FIR branch be $X_{R0}^{i}\in R^{h\times w\times c}$ and $X_{F0}^{i}\in R^{h\times w\times c}$, respectively, where *c*, *h*, and *w* are the number of feature channels, height, and width of the feature, respectively. The 2D convolution operator $F_{2Dconv}$ doubles the number of channels for $X_{R0}^{i}$ and $X_{F0}^{i}$, obtaining the expanded feature $X_{R1}^{i}\in R^{h\times w\times 2c}$ and $X_{F1}^{i}\in R^{h\times w\times 2c}$,
  (1) $$X_{R1}^{i}=F_{2Dconv}\left(X_{R0}^{i}\right),X_{R1}^{i}\left(j\right)\in R^{h\times w},j\in \left[1,2c\right]$$
  (2) $$X_{F1}^{i}=F_{2Dconv}\left(X_{F0}^{i}\right),X_{F1}^{i}\left(j\right)\in R^{h\times w},j\in \left[1,2c\right]$$
(2) Channel feature fusion module: The CFF module is shown in the green section of Figure 3. The CFF module is used to generate channel fusion features. The specific process is as follows: we use the sigmoid function $F_{sig}$ to generate weight values $W_{F1}^{i}$ FIR features $X_{F1}^{i}$, and use these weight values to multiply with RGB features $X_{R1}^{i}$ to generate channel fusion features $X_{CF}^{i}$,
  (3) $$W_{F1}^{i}\left(j\right)=F_{sig}\left(X_{F1}^{i}\left(j\right)\right),W_{F1}^{i}\in R^{h\times w},j\in \left[1,2c\right]$$
  (4) $$X_{CF}^{i}\left(j\right)=W_{F1}^{i}\left(j\right)\circ X_{R1}^{i}\left(j\right),X_{CF}^{i}\left(j\right)\in R^{h\times w},j\in \left[1,2c\right]$$
  where $W_{F1}^{i}$ is the spatial importance weight matrix of channel *j*, $X_{CF}^{i}$ is the fusion feature matrix of channel *j*, and ∘ denotes the Hadamard product.
(3) Feature down-sampling module: The FDS module is shown in the blue section of Figure 3. The FDS module is used to generate the same spatial resolution as the *i*+1th Resblockbody feature. The specific process is as follows: A 2×2 maximum pooling operator $F_{MP}$ for channel fused feature $X_{CF}^{i}$ performs local maximum pooling to output feature map $Y_{MP}^{i}$, reduced to half the resolution of the feature map to obtain features with the same resolution as the output feature of the *i*+1th Resblockbody,
  (5) $$Y_{MP}^{i}\left(j\right)=F_{MP}\left(X_{CF}^{i}\left(j\right)\right),Y_{MP}^{i}\left(j\right)\in R^{\frac{h}{2}\times \frac{w}{2}},j\in \left[1,2c\right]$$
  where $Y_{MP}^{i}\left(j\right)$ is the down-sampled fusion feature map of channel *j*.
(4) Spatial weight generation module: The SWG module is shown in the skin section of Figure 3. The SWG module generates spatial importance weight values for the RGB and FIR channel features of the *i*+1th Resblockbody. The specific process is as follows: A sigmoid function $F_{sig}$ is applied to normalize down-sampled fusion feature map $Y_{MP}^{i}\left(j\right)$ to generate a feature space importance weight matrix $W_{c}^{i}$, where
  (6) $$W_{c}^{i}\left(j\right)=F_{sig}\left(Y_{MP}^{i}\left(j\right)\right),W_{c}^{i}\left(j\right)\in R^{\frac{h}{2}\times \frac{w}{2}},j\in \left[1,2c\right]$$

Then, the spatial importance weight matrix $W_{c}^{i}$ is used to optimize the original feature $X_{R0}^{i+1}$ and $X_{F0}^{i+1}$ output from the Resblockbody (*i* + 1) nodes of the RGB and FIR branch network and obtain first-order optimized features:

(7) $$X_{R1}^{i+1}\left(j\right)=W_{c}^{i}\left(j\right)\circ X_{R0}^{i+1}\left(j\right),X_{R1}^{i+1}\left(j\right)\in R^{\frac{h}{2}\times \frac{w}{2}},j\in \left[1,2c\right]$$

(8) $$X_{F1}^{i+1}\left(j\right)=W_{c}^{i}\left(j\right)\circ X_{F0}^{i+1}\left(j\right),X_{F1}^{i+1}\left(j\right)\in R^{\frac{h}{2}\times \frac{w}{2}},j\in \left[1,2c\right]$$

where $X_{R1}^{i+1}\left(j\right)$ represents the first-order optimized feature map of Resblockbody (*i* + 1) node channel *j* in the RGB branch network; $X_{F1}^{i+1}\left(j\right)$ represents the first-order optimized feature map of Resblockbody (*i* + 1) node channel *j* in the FIR branch network; and ∘ is a Hadamard product operation.

### 3.2. RGB-FIR Multimodal Feature Fusion Module

**Figure 4:**
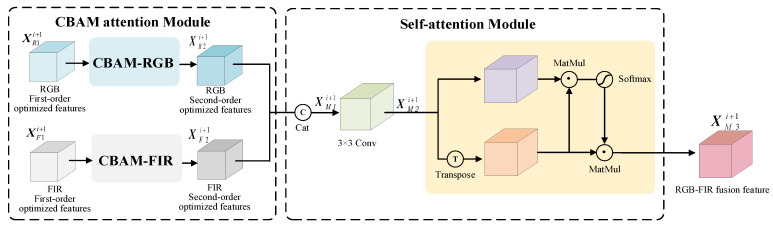
Framework of RGB-FIR multimodal feature fusion module.

The proposed RGB-FIR multimodal feature fusion module has two parts: the CBAM attention model [50] and self-attention model [51]. The network structure is shown in Figure 4. The process of feature optimization and fusion is as follows. The CBAM attention model is used to optimize the channel and space information for first-order optimized features $X_{R1}^{i+1}$ and $X_{F1}^{i+1}$ of RGB and FIR branch Resblockbody (*i* + 1), and to obtain the second-order optimized features of each branch Resblockbody (*i* + 1),

(9) $$X_{R2}^{i+1}\left(j\right)=F_{CBAM}\left(X_{R1}^{i+1}\left(j\right)\right),X_{R2}^{i+1}\left(j\right)\in R^{\frac{h}{2}\times \frac{w}{2}},j\in \left[1,2c\right]$$

(10) $$X_{F2}^{i+1}\left(j\right)=F_{CBAM}\left(X_{F1}^{i+1}\left(j\right)\right),X_{F2}^{i+1}\left(j\right)\in R^{\frac{h}{2}\times \frac{w}{2}},j\in \left[1,2c\right]$$

where $X_{R2}^{i+1}\left(j\right)$ represents the second-order optimized features of Resblockbody (*i* + 1) node channel *j* in the RGB branch CSPDarknet network, $X_{F2}^{i+1}\left(j\right)$ represents the second-order optimized features of Resblockbody (*i* + 1) node channel *j* in the FIR branch CSPDarknet network, and $F_{CBAM}$ is the CBAM attention operator.

$X_{R2}^{i+1}\left(j\right)$ and $X_{F2}^{i+1}\left(j\right)$ are used as input to construct a self-attention module for RGB-FIR multimodal feature fusion, consisting of channel concatenation, convolution, and self-attention operators, as shown in Figure 4. By using channel concatenation operators $F_{C}$, the RGB and FIR Resblockbody (*i* + 1) features $X_{R2}^{i+1}\left(j\right)$ and $X_{F2}^{i+1}\left(j\right)$ are spliced into $X_{M1}^{i+1}\left(j\right)$,

(11) $$X_{M1}^{i+1}\left(j\right)=F_{C}\left(X_{R2}^{i+1}\left(j\right),X_{F2}^{i+1}\left(j\right)\right),X_{M1}^{i+1}\left(j\right)\in R^{\frac{h}{2}\times \frac{w}{2}},j\in \left[1,4c\right]$$

where $X_{M1}^{i+1}\left(j\right)$ is the channel concatenation feature of second-order optimized features $X_{R2}^{i+1}\left(j\right)$ and $X_{F2}^{i+1}\left(j\right)$, and $F_{C}$ is the channel concatenation operators. With convolution operator $F_{conv}$, $X_{M1}^{i+1}\left(j\right)$ is compressed by channel into $X_{M2}^{i+1}$,

(12) $$X_{M2}^{i+1}=F_{conv}\left(X_{M1}^{i+1}\right),X_{M2}^{i+1}\left(j\right)\in R^{\frac{h}{2}\times \frac{w}{2}},j\in \left[1,2c\right]$$

where $F_{conv}$ represents the convolution operation of a total of 2C 3×3 convolution kernels.

The self-attention operator $F_{S}$ adjusts the weight of $X_{M2}^{i+1}$ to obtain the final RGB-FIR feature $X_{M3}^{i+1}$ of the Resblockbody (*i* + 1) node:

(13) $$X_{M3}^{i+1}\left(j\right)={\left(X_{M2}^{i+1}\left(j\right)\right)}^{T}\cdot F_{S}\left(X_{M2}^{i+1}\left(j\right)\right),X_{M3}^{i+1}\left(j\right)\in R^{\frac{h}{2}\times \frac{w}{2}},j\in \left[1,2c\right]$$

(14) $$F_{S}\left(X_{M2}^{i+1}\left(j\right)\right)=\frac{e^{\left(X_{M2}^{i+1}\left(j\right)\cdot {\left(X_{M2}^{i+1}\left(j\right)\right)}^{T}\right)}}{\sum_{1}^{T}e^{\left(X_{M2}^{i+1}\left(j\right)\cdot {\left(X_{M2}^{i+1}\left(j\right)\right)}^{T}\right)}}$$

where *T* denotes transposition, and · is matrix multiplication.

**Figure 5:**
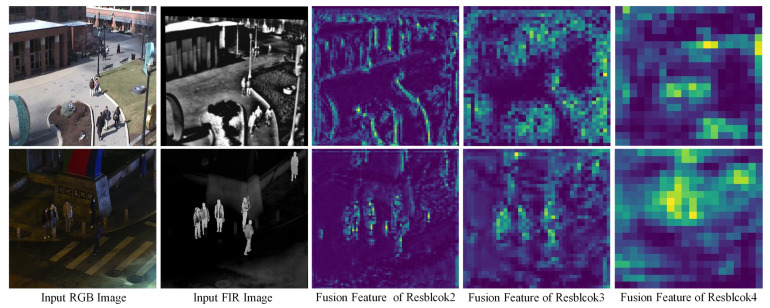
Visualization examples of fused feature output from RGB-FIR multimodal feature fusion module.

Figure 5 shows examples of RGB-FIR feature visualization at different scales for Resblockbody2, Resblockbody3, and Resblockbody4. It can be seen that, with the help of the front-end CCAM spatial weights, the backend RGB-FIR feature fusion module can provide accurate and complete pedestrian features in spatial positions.

### 3.3. Multimodal Pedestrian Detection Model YOLO-CCAM

**Figure 6:**
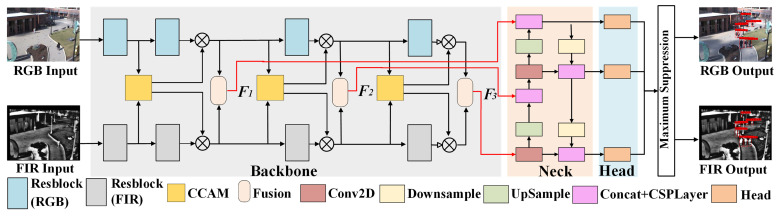
Framework of YOLO-CCAM.

Figure 6 shows the complete framework of the multimodal pedestrian detection model YOLO-CCAM, which consists of a parallel backbone network, a neck network, and a detection head. Different from existing multimodal YOLO networks, the proposed backbone network framework based on a multi-level fusion strategy is shown in the gray part of Figure 6: the front-end CCAM is used for optimizing the features of each branch, while the back-end fusion module takes optimized features as input and enhances pedestrian feature description ability through modal complementarity. The fusion features F1, F2, and F3 at three scales are used as inputs for the neck network, and multi-scale feature fusion is completed in the neck network. Finally, the multi-scale pedestrian detection results are output at the detection head. The structure of neck network and detection is the same as the original YOLOv5 model.

## 4. Experiment and Analysis

### 4.1. Experimental Setup

To establish a foundation for subsequent comparative experiments, we first introduce the experimental setup and datasets below. We combine Python (3.8) language with the PyTorch (1.11.0+cu113) framework to construct the model, using stochastic gradient descent (SGD) as the optimizer. The input image resolution is fixed at 640 × 640. The maximum number of epochs is set to 300, which ensures sufficient convergence time without overfitting, as verified by stable validation performance. The batch size is set to 4, considering the memory limitations of our hardware (NVIDIA RTX 3080, 10 GB, Nvidia: Santa Clara, CA, USA), which allows smooth training even for high-resolution inputs. The maximum learning rate is 0.02, chosen empirically based on preliminary tuning and commonly used settings in YOLO-based models to maintain stable and efficient convergence. All experiments were conducted on the same PC server equipped with an Intel Core i7-12700 2.10-GHz CPU, a 10 GB NVIDIA RTX 3080 GPU, and 64 GB of RAM (Intel, Santa Clara, CA, USA).

We used two publicly available datasets, OSU [52] and KAIST [45], to train, test, and quantitatively analyze all models. The OSU dataset contains a total of 17,089 sets of 320×240 RGB-FIR image pairs. KAIST is a multispectral pedestrian detection dataset with 95,328 sets of RGB-FIR multimodal image data, with 640×512 resolution. The dataset includes three label types: person, people, and cyclist. We used only single-person data of the first label type for training and testing.

To comprehensively evaluate the detection performance and real-time capability of each model, we employed the following metrics: Precision, Recall, mAP, Running time, *FPS*, and parameter volume to comprehensively evaluate the experimental results of all models. These are defined as follows:

(15) $$\;\mathrm{Precision}\;=\frac{TP}{TP+FP}$$

(16) $$\;\mathrm{Recall}\;=\frac{TP}{TP+FN}$$

where *TP* is the number of correctly identified positive samples, *FP* is the number of negative samples incorrectly identified as positive, and *FN* is the number of positive samples incorrectly identified as negative.

(17) $$AP=\int_{0}^{1}\mathrm{Precision}(r)dr=\int_{0}^{1}\frac{TP(r)}{TP(r)+FP(r)}dr$$

where *AP* is the area below the *Precision-6Recall* curve, *P*(*r*) denotes the precision at a given recall *r*.

(18) $$mAP=\frac{1}{n}\sum_{i=0}^{n}AP_{i}=\frac{1}{n}\int_{0}^{1}P_{i}\left(r\right)dr$$

where *mAP* is the average accuracy of all categories, representing the average *mAP* at different degree of overlap between the predicted box and the real box (0.5–0.95, step0.05).

(19) $$\;\mathrm{Running}\;\mathrm{time}\;=T_{\mathrm{end}\;}-T_{\mathrm{begin}\;}$$

(20) $$FPS=\frac{T_{\mathrm{interval}\;}}{\;\mathrm{Running}\;\mathrm{time}\;}$$

where *Running time* is the time required for the model to test 100 images, $T_{\mathrm{end}\;}$ and $T_{\mathrm{begin}\;}$ are the respective end and start times of the test, and $T_{\mathrm{interval}\;}$=100.

### 4.2. Baseline Model Comparison

**Figure 7:**
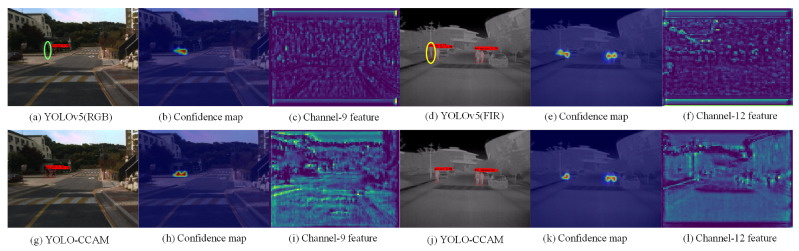
Visualization of features and confidence of different YOLO models (missing detection and false detection).

To verify the effectiveness of the proposed multimodal pedestrian detection model framework, we conducted comparative experiments on the KAIST dataset (70%:30%), comparing YOLO-CCAM with the unimodal YOLOv5 (RGB/FIR). Figure 7 shows some examples of pedestrian detection results for different YOLO models, whose first row shows detection results, confidence maps, and feature maps on a randomly selected Resblockbody2 channel features for YOLOv5(RGB/FIR) models. The second row shows the detection results, a confidence map, and a feature map on Resblockbody2 of the YOLO-CCAM. A red rectangular box represents a detected pedestrian, a green oval box a missed pedestrian target, and a yellow oval box a falsely detected pedestrian target. Through comparison, it can be seen that both RGB and FIR images exhibit pedestrian target error detection in the single-mode YOLOv5 model, as shown in Figure 7a,d. This indicates that it is difficult to extract accurate pedestrian features from YOLOv5 in complex environments using only RGB or FIR images, as shown in Figure 7c,f. As a result, the feature representation ability of pedestrian targets is weak, and it is unable to generate high confidence values for pedestrian categories, as shown in Figure 7b,e. The proposed method can improve the representation ability of pedestrian targets in complex environments through multi-level feature fusion of RGB and FIR, as shown in Figure 7i,l, enhancing the detection confidence values of targets, as shown in Figure 7h,k.

**Figure 8:**
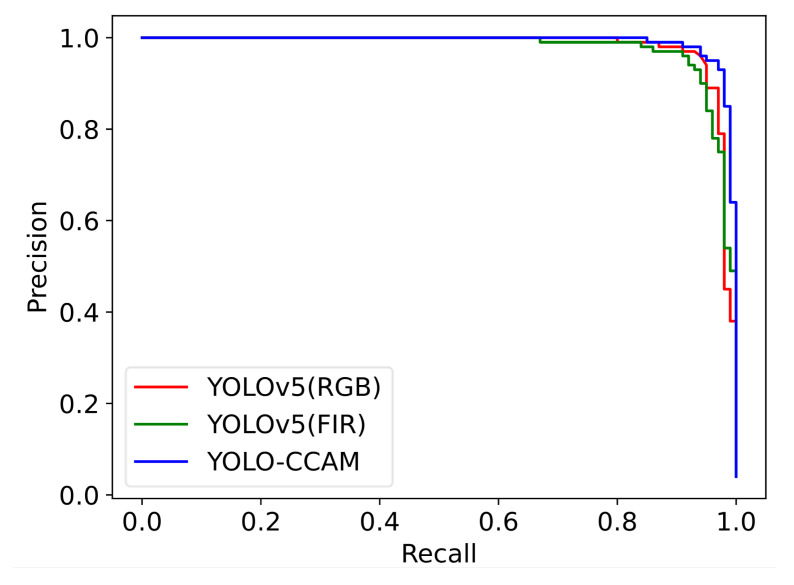
Feature and Confidence Visualization of Single-Modality vs. YOLO-CCAM Models.

To evaluate the detection performance of YOLOv5 and YOLO-CCAM under different thresholds, Figure 8 shows three Precision–Recall curves on the KAIST dataset, whose comparison shows that, when Recall is less than 0.97, the red curve of YOLOv5 (RGB) is at the upper-right of YOLOv5 (FIR), and when Recall is greater than 0.97, the green curve of YOLOv5 (FIR) is at the upper-right of the YOLOv5 (RGB) model; that is, YOLOv5 (RGB) and YOLOv5 (FIR) have advantages and disadvantages in pedestrian detection across the entire Precision–Recall coordinate system. The blue curve of YOLO-CCAM is at the upper-right of the two single-modal models, and YOLOv5 (RGB/FIR) is on the entire Precision–Recall coordinate system, regardless of the threshold. This indicates that, compared to the single-modality model, after multi-level optimization and feature fusion, YOLO-CCAM can provide more accurate pedestrian detection results. Further investigation will focus on optimal feature enhancement and fusion strategies.

### 4.3. Effectiveness of the CCAM Module

The proposed CCAM belongs to the attention mechanism module, and the essential difference between it and the existing feature fusion modules for RGB-FIR multimodal object detection [19,20,21] lies in the module’s usage position and role. The CCAM is embedded at the front end of the parallel backbone network, as shown by the yellow box in Figure 1b, and its output weight values are used to optimize the channel feature maps of the RGB and FIR branches of the parallel backbone network. The existing multimodal feature fusion modules [19,20,21] are all embedded in the backend of the parallel backbone network, as shown in the skin color module in Figure 1a. These RGB-FIR fusion features F1, F2, and F3 output by them are used for feature fusion in the neck network, and they do not have the role of optimizing the features of the RGB and FIR branches of the backbone network.

To demonstrate the effectiveness of CCAM compared to existing methods [19,20,21], the comparative experiments were conducted on the KAIST dataset for two types of different multimodal backbone networks. The first type framework is shown in Figure 1a, including CSPDarknet (RGB, FIR) + Fusion [19], CSPDarknet (RGB, FIR) + Fusion [20] and CSPDarknet (RGB, FIR) + Fusion [21]. The second type framework is shown in Figure 1b, including CSPDarknet (RGB, FIR) + CCAM + Fusion [19], CSPDarknet (RGB, FIR) + CCAM + Fusion [20], and CSPDarknet (RGB, FIR) + CCAM + fusion [21].

**Figure 9:**
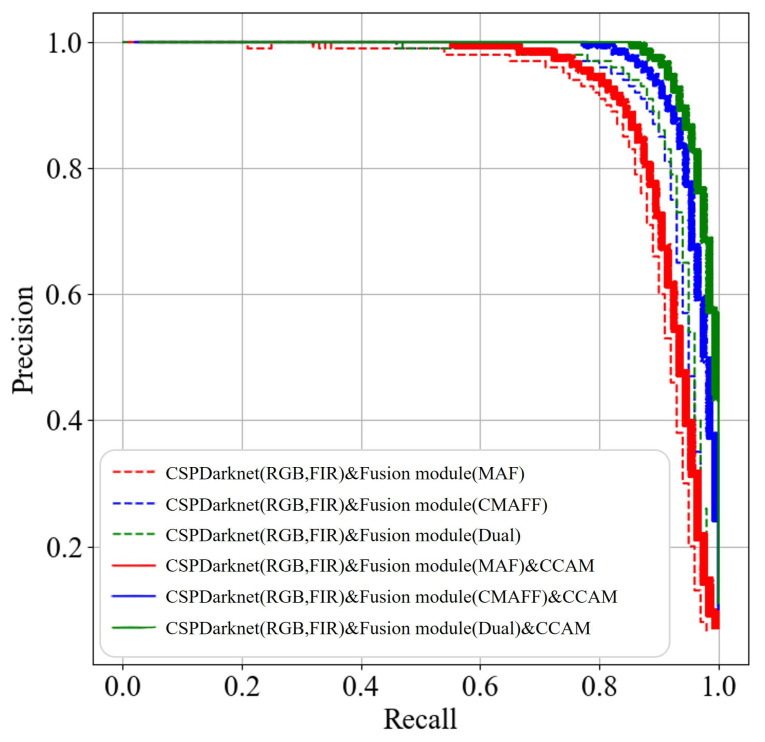
Precision–Recall curve of the CCAM effectiveness (KAIST) [19,20,21].

Figure 9 shows the Precision–Recall curves of the above two categories of YOLO backbone network structures under various detection thresholds on the KAIST dataset. Comparing the positional relationships of different colored curves, the Precision–Recall curves of those multimodal YOLO backbone networks with CCAMs are clearly located in the upper-right corner of those multimodal YOLO backbone networks without the CCAM modules. That is, regardless of which fusion module is selected at back-end, the detection performance of the multimodal YOLO backbone network with the addition of the CCAM module at front-end has been significantly improved.

Moreover, to further quantify the effectiveness of CCAM, Table 1 shows the *mAP* evaluation results of above two types of multimodal YOLO backbone network frameworks. Obviously, CCAM increased on average *mAP0.5*, *mAP0.75*, and *mAP* by 2.93%, 3.53%, and 4.76%, respectively. This indicates that, under the optimization of front-end CCAM, the backbone network framework based on multi-level fusion strategy can provide more effective pedestrian description features.

To obtain the optimal structure of CCAM, we constructed three different structures: $CCAM_{\mathrm{Multiple}}$, $CCAM_{\mathrm{MAX}}$, and $CCAM_{\mathrm{SUM}}$, respectively. The main difference lies in the fusion method between RGB and FIR features after channel expansion. $CCAM_{\mathrm{Multiple}}$ represents the operation of normalizing FIR feature values using the sigmoid function and multiplying them with RGB feature values. $CCAM_{\mathrm{MAX}}$ represents the operation of selecting the maximum value between the corresponding RGB and FIR features. $CCAM_{\mathrm{SUM}}$ represents the addition operation of corresponding RGB and FIR features.

Table 2 evaluates $CCAM_{\mathrm{Multiple}}$, $CCAM_{\mathrm{MAX}}$, and $CCAM_{\mathrm{SUM}}$ at pedestrian target detection on the OSU dataset (70% training, 30% testing) using *mAP0.5*, *mAP0.75*, and *mAP*. It can be seen that, compared to $CCAM_{\mathrm{MAX}}$, $CCAM_{\mathrm{SUM}}$ increased by 0.7% on *mAP0.5*, 1.2% on *mAP0.75*, and 3.4% on *mAP*. Compared to $CCAM_{\mathrm{SUM}}$, $CCAM_{\mathrm{Multiple}}$ increased by 0.1% on *mAP0.5*, 1.8% on *mAP0.75*, and 1.1% on *mAP*. This indicates that the fusion method for adjusting RGB features using FIR features normalized by a sigmoid function as weight values is superior to the two fusion methods of sum and maximum.

### 4.4. Comparison with Other Multimodal Models

To illustrate the advantages of the proposed YOLO-CCAM compared to existing multimodal YOLO models, we compared it with the most recent RGB-FIR multimodal YOLO pedestrian detection models, including YOLO-CMAFF [19], Dual YOLO [20], and MAF-YOLO [21]. We trained and tested all models on the OSU and KAIST public datasets, with 70% for training and 30% for testing, based on *mAP0.5*, *mAP0.75*, *mAP*, the Precision–Recall curve, *Time*, *FPS*, and *Para*.

**Figure 10:**
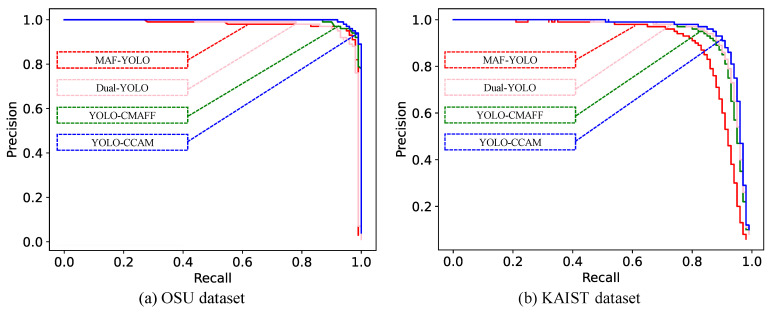
Precision–Recall performance of different multimodal YOLO pedestrian detection models on OSU and KAIST dataset.

Figure 10 shows the Precision–Recall curves of four RGB-FIR multimodal YOLO pedestrian detection models on the OSU and KASIT datasets. Through comparison, we can see that the blue curve of YOLO-CCAM is in the upper-right corner of the Precision–Recall curve on both datasets of other methods. This indicates that, regardless of the detection threshold setting, the performance of the YOLO-CCAM model based multi-level fusion strategy is better than that of existing RGB-FIR multimodal YOLO networks based on back-end fusion strategies.

**Figure 11:**
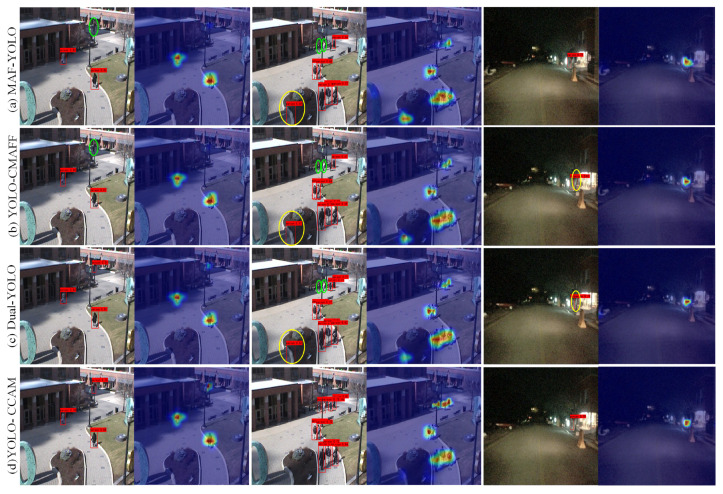
Detection Confidence and Error Comparison among Multimodal YOLO Models.

Figure 11 shows the detection results and confidence maps of the four models. Through comparison, we can see that MAF-YOLO and YOLO-CMAFF have missed detections of remote small targets on both datasets, as shown in the green ellipses in Figure 11. This indicates that the RGB-FIR multimodal fusion module in these models cannot provide accurate feature representation capabilities for small pedestrians in complex environments, resulting in low confidence values for pedestrian categories, as shown in the pedestrian confidence thermal maps in Figure 11. Dual-YOLO has a higher confidence level at pedestrian detection due to its multiscale inception feature extraction unit in the RGB-FIR multimodal feature fusion module. MAF-YOLO, YOLO-CMAFF, and Dual-YOLO all experience false positives, as shown in the yellow ovals in Figure 11. This indicates that the effectiveness of RGB-FIR multimodal backbone networks based on back-end fusion strategies largely depends on the effectiveness of the raw RGB and FIR features in the front-end. If the original RGB and FIR features of the front-end cannot effectively distinguish similar non-pedestrian targets, it is difficult for the RGB-FIR fusion features of the back-end to improve the representation ability of the features; this results in high confidence in the pedestrian category of similar object positions, as shown in the confidence maps in Figure 11. YOLO-CCAM based on the multi-level fusion strategy, with the help of front-end CCAM and back-end RGB-FIR multimodal fusion modules, exhibits more accurate pedestrian detection confidence values and detection results compared to the other three models, as shown in Figure 11d.

To validate the effectiveness of the proposed YOLO-CCAM model in pedestrian detection applications, 13 kinds of pedestrian detection models were selected. Among them, SSD [39], YOLOv3 [23], YOLOv4 [24], YOLOv5 [18], and YOLOv7 [25] are single-modality models, and YOLO-CMAFF [19], Dual-YOLO [20], and MAF-YOLO [21] are RGB-FIR multimodal models. The comparison models were trained and tested on the OSU and KASIT datasets, as was the proposed model, and all the experiments were conducted in the same environment. Table 3 shows the values of *mAP0.5*, *mAP0.75*, *mAP*, *Time (ms)*, *FPS*, and *Para (M)* for all methods.

If the accuracy of 10 single modal pedestrian detection models is analyzed separately, on the OSU dataset, the *mAP0.5* of YOLOv3 (RGB) reaches 95.5%, the *mAP0.75* of YOLOv7 (RGB) reaches 65.8%, and the *mAP* of YOLOv5 (RGB) reaches 56%. The accuracy of these three models is higher than that of other models; On the KAIST dataset, the *mAP0.5* of YOLOv3 (FIR) reached 90.5%, the *mAP0.75* of YOLOv5 (RGB) reached 59.5%, and the *mAP* of YOLOv7 (FIR) reached 53.4%. These three models have higher accuracy than other models.

If we analyze the above 10 single-mode pedestrian detection models from the perspectives of real-time detection, image processing speed, and model parameter volume, YOLOV5 is the fastest running model, with the strongest image processing ability, and smallest parameter volume among the 10 single-modality models on the OSU and KAIST datasets. *Time* reached 11.5 ms, *FPS* 90.4 frames per second, and *Para* 7.1M. From the above analysis, it can be inferred that, among the 10 single-modality models, YOLOv7 has the highest detection accuracy and is suitable for server environments or cloud computing applications; YOLOv5 has the fastest processing speed and smallest parameter volume, making it more suitable for applications in embedded environments.

From the accuracy comparison of the two types of pedestrian detection models (single-modality and multimodal) shown in Table 3, it can be observed that the accuracy of the four RGB-FIR multimodal pedestrian detection models on the OSU and KAIST datasets is higher than that of the 10 single-modality models. Among them, YOLO-CCAM has the highest *mAP0.5* and *mAP* on the OSU dataset, reaching 97.2% and 56.9%, respectively. On the KAIST dataset, YOLO-CCAM has the highest *mAP0.5*, *mAP0.75*, and *mAP*, of 97.2%, 60.8%, and 56.9%, respectively.

The phenomenon that the RGB-FIR multimodal detection model outperforms the single-modality pedestrian detection model in accuracy confirms the hope that the method of adding human features through the modal complementarity of RGB and FIR images is correct and feasible. Moreover, the effectiveness of the RGB-FIR multimodal backbone network framework based on front- and back-end multi-level fusion strategies for pedestrian feature enhancement in this paper is significantly better than the existing RGB-FIR multimodal backbone network framework design based on back-end fusion strategies [19,20,21].

However, while utilizing parallel backbone networks to improve detection accuracy, the four RGB-FIR multimodal detection models show some increase in parameter volume and decrease in runtime and processing speed, and compared to YOLOv5. *Time* of MAF-YOLO increases to 48.4ms, *FPS* decreases to 20.9 frames per second, and *Para* increases to 32.6M. *Time* of YOLO-CMAFF increases to 21.7 ms, *FPS* decreases to 48.1 frames per second, and *Para* increases to 13M. *Time* of Dual-YOLO increases to 25.1 ms, *FPS* decreases to 40.4 frames per second, and *Para* increases to 19.6M. The *Time* of YOLO-CCAM increases to 23.8 ms, *FPS* decreases to 42.7 frames per second, and *Para* increases to 21.2M.

From the comprehensive evaluation indicators of accuracy and real-time performance in Table 3, among the 13 models presented, the proposed YOLO-CCAM achieves the best detection performance in terms of accuracy on both the OSU and KAIST datasets. At the same time, in terms of real-time performance, YOLO-CCAM’s runtime and parameter volume cloud are superior to the latest single-mode detection model, YOLOv7. Therefore, the YOLO-CAM model has good accuracy and real-time performance in pedestrian detection tasks in server or cloud computing environments.

**Figure 12:**
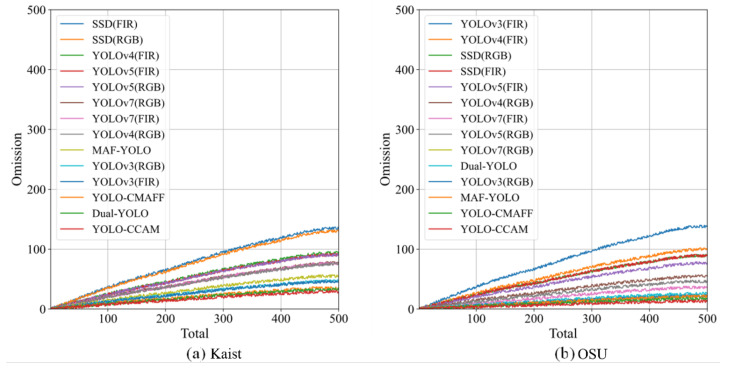
Omission rate performance of all the pedestrian detection models on test datasets. (**a**) KAIST dataset, (**b**) OSU dataset.

Moreover, an omission rate is used to evaluate the robustness of proposed YOLO-CCAM model in this paper. The omission rate represents the unreliability of the network structure. In the test set of the public Kaist and OSU dataset, 500 pictures are stochastically chosen for the experiments of the omission rate, respectively. The experimental results of all 14 pedestrian detection models mentioned above are shown in Figure 12a,b. The comparably lower omission rate of YOLO-CCAM corroborates its higher robustness compared to the existing methods in pedestrian detection fields.

### 4.5. Real-World Pedestrian Detection Application

To further evaluate the generalization ability and performance of the proposed CCAM module, we conducted additional experiments on the LLVIP dataset, a widely used thermal-visible pedestrian detection benchmark [53]. The dataset contains 12,025 training samples and 3,464 testing samples, with paired RGB and FIR images captured under varying illumination conditions, making it suitable for evaluating vision models in both day and night scenarios. In addition to the comparison with YOLOv7, this study also introduces three more recent detection frameworks: YOLOv9 [54], YOLOv11 [55], and YOLOv12 [56]. All comparison models are evaluated under a single-modality setting, with independent experiments conducted using RGB and FIR inputs. As shown in Table 4, YOLOv9(FIR) achieves the best detection performance among all models, with 96.40% mAP0.5, 74.70% mAP0.75, and 65.60% mAP, outperforming both YOLOv11(FIR) and YOLOv12(FIR). Based on the YOLOv9 architecture, we integrated the proposed CCAM module to construct YOLOv9-CCAM, which improves the overall mAP by 0.2%, reaching 65.80%. These results demonstrate the effectiveness of the proposed CCAM module in enhancing spatial–semantic feature representation.

**Figure 13:**
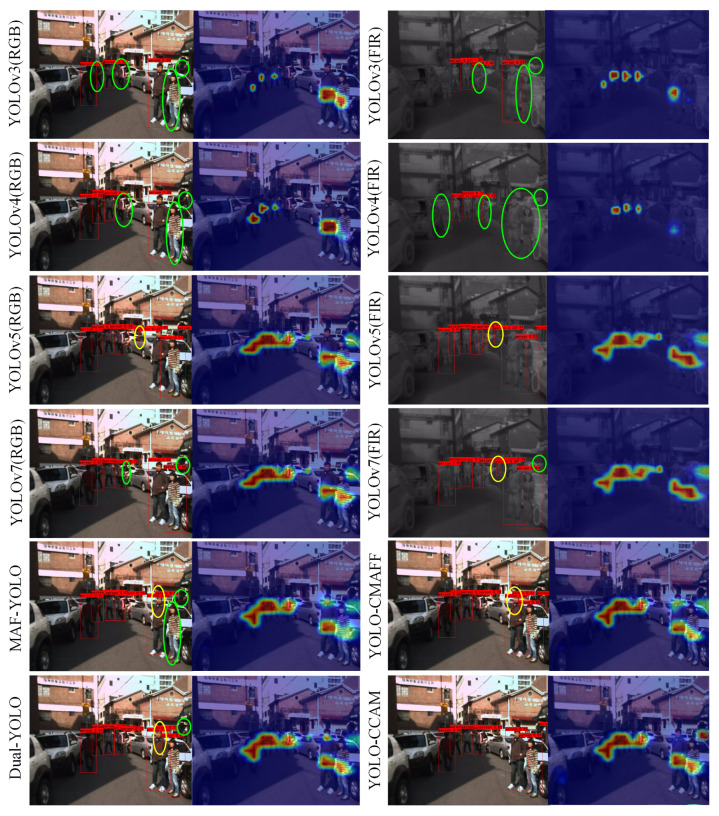
Performance examples of different pedestrian detection models in the KAIST dataset.

Figure 13 shows an example of pedestrian detection results and confidence visualization for 12 models on the KAIST dataset, where the first row provides the pedestrian detection results and corresponding pedestrian target confidence thermal maps of the YOLOv3 in RGB and FIR images, respectively. The second row provides pedestrian detection results and corresponding pedestrian target confidence thermal maps of the YOLOv4 in RGB and FIR images, respectively. The third row provides pedestrian detection results and corresponding pedestrian target confidence thermal maps of the YOLOv5 in RGB and FIR images, respectively. The fourth row provides pedestrian detection results and corresponding pedestrian target confidence thermal maps of the YOLOv7 in RGB and FIR images, respectively.

The first two images in the fifth row of Figure 13 provide the pedestrian detection results and corresponding pedestrian target confidence thermal maps of MAF-YOLO under the same set of RGB FIR multimodal image pairs, and the last two images provide the pedestrian detection results and corresponding pedestrian target confidence thermal maps of the YOLO-CMAFF model under the same set of RGB-FIR multimodal image pairs. The first two images in the sixth row provide pedestrian detection results and corresponding pedestrian target confidence thermal maps of the Dual-YOLO model under the same set of RGB FIR multimodal image pairs. The last two images in the fifth row provide pedestrian detection results and corresponding pedestrian target confidence thermal maps of the YOLO-CCAM model under the same set of RGB-FIR multimodal image pairs.

Among them, a red box represents pedestrian detection results, a green ellipse represents a missed pedestrian target, and a yellow ellipse represents a mistakenly detected pedestrian target. Through comparison, it can be seen that the confidence map generated by the proposed YOLO-CCAM can more accurately represent the position of pedestrians than those of other models.

## 5. Conclusions

Here, we focused on an RGB-FIR multimodal pedestrian detection model, organizing the latest construction method and characteristics of a multimodal YOLO backbone network framework based on back-end fusion. To address the shortcomings of existing multimodal YOLO backbone network back-end fusion frameworks, an RGB-FIR multimodal YOLO backbone network framework based on a CCAM multi-level fusion strategy was proposed. Starting from the original feature extraction module of the backbone network, the spatial weight values of the upper RGB and FIR modal features were optimized by prior knowledge with the lower RGB-FIR fusion features. We implemented cross-modal and cross-scale fusion for various modal and channel features. Furthermore, we prevented local effective features at the bottom from drifting or disappearing with the deepening of network layers and the increase in the receptive field. After comparing and evaluating the accuracy, robustness, real-time performance, and parameter volume of pedestrian target detection models on two public datasets, the proposed YOLO-CCAM model was found to effectively enhance the accuracy of pedestrian target detection while maintaining good real-time image processing capabilities.

In future work, we plan to further expand our model to support additional modalities, such as depth information, to enhance perception in complex environments. Moreover, adaptive fusion strategies that dynamically adjust to varying scene contexts will be explored to further improve detection robustness and generalization.

## Figures and Tables

**Table 1:** Effectiveness of ccam module (KAIST).

Backbone Network Type	mAP0.5	mAP0.75	mAP
CSPDarknet (RGB, FIR) and Fusion [19]	91.30%	55.10%	51.90%
CSPDarknet (RGB, FIR) and Fusion [19] and CCAM	93.80%	58.30%	55.40%
CSPDarknet (RGB, FIR) and Fusion [20]	92.70%	57.10%	54.20%
CSPDarknet (RGB, FIR) and Fusion [20] and CCAM	94.90%	59.80%	57.20%
CSPDarknet (RGB, FIR) and Fusion [21]	88.30%	46.00%	46.00%
CSPDarknet (RGB, FIR) and Fusion [21] and CCAM	92.40%	56.60%	53.30%

**Table 2:** Performance comparison of CCAM fusion strategies.

Model	mAP0.5	mAP0.75	mAP
${\mathrm{CCAM}}_{\mathrm{MAX}}$	96.40%	58.40%	52.40%
${\mathrm{CCAM}}_{\mathrm{SUM}}$	97.10%	59.20%	55.80%
${\mathrm{CCAM}}_{\mathrm{Multiple}}$	97.20%	61.00%	56.90%

**Table 3:** Comparison results of pedestrian detection models on the OSU dataset.

Mono-modality pedestrian detection models									
Model	OSU			KAIST			ALL		
	mAP0.5	mAP0.75	mAP	mAP0.5	mAP0.75	mAP	Time	FPS	Para
SSD (RGB) [39]	82.00%	60.50%	51.90%	73.90%	55.60%	48.60%	67.3	14.9	23.8
SSD (FIR) [39]	82.00%	63.90%	52.80%	72.90%	54.30%	48.00%			
YOLOv3 (RGB) [23]	95.50%	63.00%	55.60%	90.50%	55.90%	52.60%	18.6	55.5	61.5
YOLOv3 (FIR) [23]	72.20%	23.80%	33.20%	90.70%	54.10%	52.50%			
YOLOv4 (RGB) [24]	88.90%	42.80%	46.70%	84.90%	57.90%	52.40%	22.7	46.1	63.9
YOLOv4 (FIR) [24]	79.90%	38.20%	41.20%	81.10%	38.70%	42.50%			
YOLOv5 (RGB) [18]	90.60%	63.80%	56%	81.80%	59.50%	52.30%	11.5	90.4	7.1
YOLOv5 (FIR) [18]	84.50%	56.90%	50.30%	81.70%	58.60%	52.30%			
YOLOv7 (RGB) [25]	95.40%	65.80%	55.90%	84.50%	58.80%	53.10%	17.3	57.8	37.2
YOLOv7 (FIR) [25]	92.60%	63.10%	55.20%	84.60%	59.00%	53.40%			
Multi-modality pedestrian detection models									
Model	OSU			KAIST			ALL		
	mAP0.5	mAP0.75	mAP	mAP0.5	mAP0.75	mAP	Time	FPS	Para
MAF-YOLO [21]	95.70%	48.20%	51.10%	88.80%	46.40%	47.80%	48.4	20.9	32.6
YOLO-CMAFF [19]	96.40%	56.10%	54.80%	92.80%	55.90%	53.90%	21.7	48.1	13
Dual-YOLO [20]	94.70%	53.70%	53.70%	93.30%	57.80%	54.80%	25.1	40.4	19.6
YOLO-CCAM	97.20%	61.00%	56.90%	94.10%	60.80%	56.50%	23.8	42.7	21.2

**Table 4:** Prediction accuracy comparison with the latest YOLO framework on the LLVIP dataset.

Model	mAP0.5	mAP0.75	mAP
YOLOv9 (RGB)	90.60%	56.20%	52.40%
YOLOv9 (FIR)	96.40%	74.70%	65.60%
YOLOv11 (RGB)	88.40%	50.00%	49.20%
YOLOv11 (FIR)	96.30%	73.60%	64.60%
YOLOv12 (RGB)	89.00%	50.30%	49.50%
YOLOv12 (FIR)	96.00%	73.90%	64.50%
YOLO-CCAM	96.70%	73.20%	64.90%
YOLOv9-CCAM	97.10%	75.10%	65.80%

## Data Availability

The datasets used and/or analyzed during the current study are available from the corresponding author upon reasonable request.
